# Collagen-derived peptide, DGEA, inhibits pro-inflammatory macrophages in biofunctional hydrogels

**DOI:** 10.1557/s43578-021-00423-y

**Published:** 2021-12-02

**Authors:** Aakanksha Jha, Erika Moore

**Affiliations:** 1 J. Crayton Pruitt Family Department of Biomedical Engineering, Herbert Wertheim College of Engineering, University of Florida, Gainesville, FL 32611 USA; 2 Department of Materials Science and Engineering, Herbert Wertheim College of Engineering, University of Florida, Gainesville, FL 32611 USA

**Keywords:** Collagen-derived peptide, Macrophages, Hydrogel, Inflammation, Biomaterial

## Abstract

Macrophages are innate immune cells that play important roles in wound healing. Particularly, M1 macrophages are considered pro‐inflammatory and promote initial phases of inflammation. Long-term exposure to inflammatory stimuli causes an increase in M1 macrophages, which contributes to chronic inflammation. Activated M1 macrophages have been shown to upregulate integrin α2β1 expression. To interfere with α2β1 binding, we designed a biofunctional hydrogel utilizing a collagen I-derived peptide, DGEA (Asp-Gly-Glu-Ala). We hypothesize that M1 macrophage activation can be reduced in the presence of DGEA. Effects of DGEA on M1 macrophages were studied via soluble delivery and immobilization within poly(ethylene glycol) (PEG) hydrogels. We demonstrate that M1 macrophage activation is reduced both via soluble delivery of DGEA in 2D and via immobilized DGEA in a 3D PEG-DGEA hydrogel. This novel biomaterial can manipulate inflammatory macrophage activation and can be applied to prevent chronic inflammatory conditions via macrophage manipulation.

**Graphical abstract:**

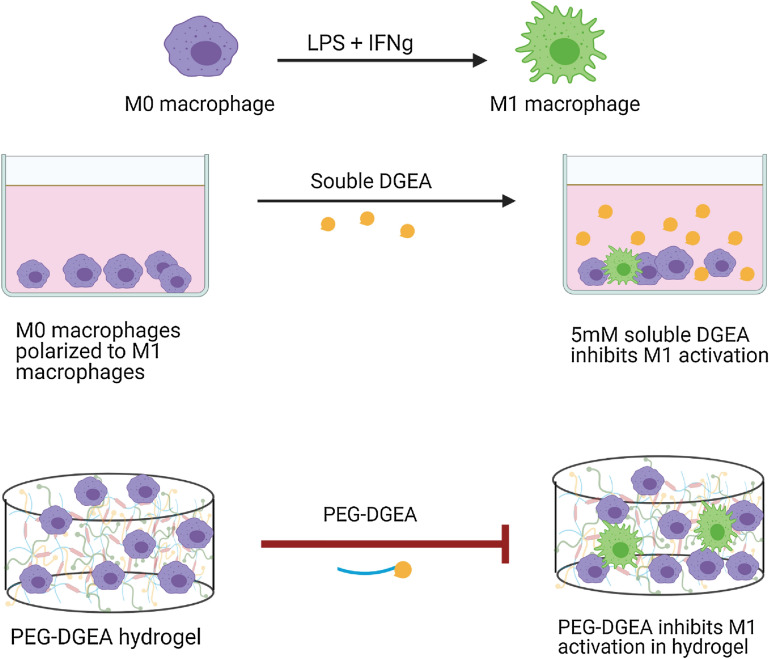

## Introduction

Inflammation is the body’s defense mechanism in response to injurious stimuli such as damaged cells or pathogens [1]. Inflammation initiates wound healing as the first stage of the immune response. In situations where inflammation persists, a healthy wound healing cascade may transition to a chronic inflammatory disorder. Throughout the world, 3 out of 5 people die due to chronic inflammatory conditions such as cardiovascular and pulmonary diseases, osteoarthritis, and diabetes [2]. Thus, there is a dire need to control inflammation through manipulation of the immune response.

Macrophages are immune cells integral to the promotion of wound healing and resolution of inflammation [3]. Macrophages are highly plastic cells that change their functions based on environmental cues, thereby acquiring various phenotypes. Macrophage polarization, or plasticity, towards different phenotypes allows macrophages to adopt various functional states. For the purposes of this work, we will use macrophage polarization to denote alterations in macrophage state [4]. Unstimulated macrophages are typically termed M0 macrophages. M1 macrophages, also called “classically activated,” are associated with a pro-inflammatory phenotype [5]. They express pro-inflammatory cytokines such as tumor necrosis factor (TNF)-α and interferon (IFN)-γ [6, 7]. To promote inflammation, M1 macrophages express inducible nitric oxide synthase (iNOS) which has antimicrobial effects towards pathogens. iNOS is a hallmark marker for M1 macrophages as its upregulation is induced by a hypoxic environment, pro-inflammatory cytokines (IFNγ, TNFα), as well as microbial factors such as lipopolysaccharide (LPS) [7]. M2, or “alternatively activated” macrophages, are an inflammation-resolution phenotype that express anti-inflammatory cytokines. When macrophages are exposed to inflammatory stimuli during the wound healing cascade, they release cytokines that initiate inflammation. A disproportionate production of inflammatory cytokines causes an excess of the M1 macrophages [7, 8]. The dysregulation of the M1 macrophage population density at a site of inflammation or wound healing can contribute to chronic inflammation and promote chronic inflammatory disorders [8, 9]. Interventions which therapeutically repolarize macrophages, in the form of preventing the M1 phenotype for example, can be beneficial for treatment of chronic inflammatory diseases.

Extracellular matrix (ECM) proteins also inform macrophage polarization. Collagen and other ECM proteins such as fibrin or laminin have often been used as biomaterials to assess cell function. To offer manipulation of the microenvironments, ECM-derived peptides such as RGDS found in fibronectin, collagen IV-derived GFPGER, and laminin-derived IKVAV, are utilized to alter cellular responses via integrin receptor interactions [10]. Specifically, the α2β1 integrin receptor interacts with Type I collagen and mediates extracellular signals to macrophages [11–15]. Integrin α2β1 appears to play a pivotal role in macrophage polarization by affecting downstream signaling pathways [14]. A study by Cha et al. showed that binding via integrin α2β1 strongly increased CD86 (M1 marker) expression and reduced CD206 (M2 marker) expression in gelatin methacryloyl (GelMA) and poly (ethylene glycol) diacrylate (PEGDA) hydrogels, whereas blocking the binding sites of α2β1 via DGEA-coated plates led to a higher expression of CD206 (M2 marker).

With focus on the function of α2β1 in macrophage polarization, we analyzed the role of DGEA in macrophage manipulation via ECM-peptide interaction. DGEA is a tetrapeptide of the sequence Asp-Gly-Glu-Ala which corresponds to residues 435–438 of the Type I collagen sequence [11]. Staatz et al. synthesized peptides, 12–13 amino acids in length, to assess inhibition of platelet adhesion to collagen. Particularly, the α2β1 integrin receptor was chosen due to the overlap between collagen and laminin recognition. After assessing the importance of aspartic acid and glutamic acid in adhesion of cells, it was concluded that DGEA is the minimal tetrapeptide recognition sequence for α2β1. In a study by Mizuno et al. [13], collagen–integrin interaction was interrupted by addition of the DGEA peptide to the culture. Fishman et al. have also demonstrated DGEA to be effective in blocking adhesion to collagen via α2β1 integrin mediation [16]. This work demonstrates that DGEA can block the binding sites of the α2β1 integrin. Thus, from previous work which shows both that α2β1 is associated with upregulation of M1 macrophages and that DGEA blocks α2β1, we hypothesized that the presence of DGEA can reduce the M1 macrophage phenotype, suggesting DGEA as a potential inhibitor for M1 macrophage polarization.

To mitigate the induction of pro-inflammatory M1 macrophages, we studied the macrophage response to DGEA both in a soluble form and in an immobilized hydrogel using polyethylene glycol (PEG) conjugated with DGEA. A previous study by Wu et al. has demonstrated immobilization of DGEA in a hydrogel to investigate adhesion of valve interstitial cells via integrins [17]. Other studies have utilized PEG-DGEA to better understand effects of shear and tension in tenocytes, cells of the tendon [18], and to model tissue-specific regeneration of endothelial cells and keratocytes [19]. The work by Cha et al. was instrumental in establishing a relationship between macrophage phenotype modulation via integrin mediation [14]. To our knowledge, immobilized DGEA has not been used to manipulate macrophage phenotype beyond this work. Our results showed that DGEA can reduce M1 polarization via both 2D soluble delivery in the media and immobilized with PEG as PEG-DGEA. This novel work demonstrates the use of DGEA, a collagen-derived peptide, to influence macrophage phenotype. Our design can be used to manipulate pro-inflammatory macrophage polarization and can be employed as a biomaterial tool to address chronic inflammatory diseases.

## Results and discussion

Non-resolving, persistent inflammation contributes to wound chronicity leading to a myriad of ailments. Macrophage polarization can guide the transition of the immune response away from inflammation towards regeneration [15]. Macrophage polarization can be engineered by designing immunomodulating biomaterials. In this work, we introduce a novel biomaterial design using a collagen-derived peptide, DGEA, which can inhibit M1 macrophage polarization. To our knowledge, no previous studies show inhibition in M1 polarization by using this collagen-derived peptide. This work is novel because DGEA is used as a soluble factor in 2D cultures, as well as covalently bound with PEG in 3D hydrogels, to successfully interfere with M1 macrophage phenotype.

### Soluble delivery of DGEA to 2D cultures of macrophages

**Figure 1:**
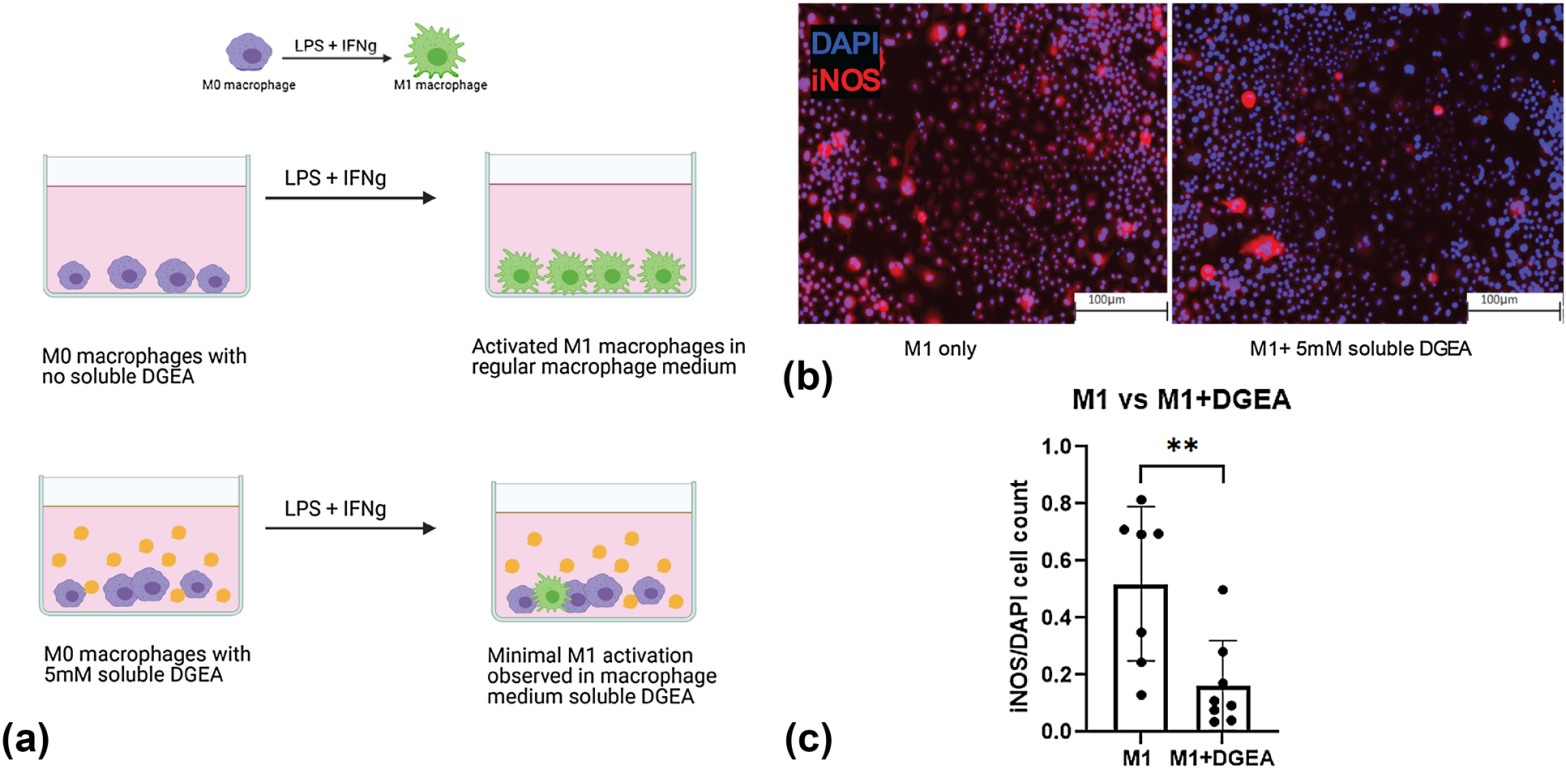
Soluble delivery of DGEA to 2D cultures. (a) M0 macrophages were polarized to the M1 phenotype by M1 media. 5mM soluble DGEA was added to media in experimental condition. (b) Immunofluorescent images of cells stained for DAPI and iNOS with (left) no DGEA and (right) 5 mM soluble DGEA. (c) Graph representing number of iNOS^+^ cells normalized to DAPI^+^ cells in control conditions (*n* = 7) and in samples with 5 mM DGEA (*n* = 8). Data represent statistical significance (*p* < 0.05) via student’s *t* test.

To establish interactions between DGEA and M1 macrophages, we conducted a 2D study with soluble delivery of DGEA to the media culture. Macrophages were stimulated to the M1 phenotype by addition of LPS and IFNγ as can be seen in Fig. 1(a). 5 mM DGEA was dissolved in the media to assess the effects of DGEA on macrophage polarization [20]. After stimulation, the cells were stained for 4′,6-diamidino-2-phenylindole (DAPI) and iNOS (Fig. 1(b)). The addition of 5 mM DGEA in the media reduced the number of iNOS^+^ cells, as represented in Fig. 1(b, c). The iNOS^+^ cell count was normalized to the number of DAPI^+^ cells for each image chosen to be analyzed. Performing a Student’s *t* test on the data demonstrated that the conditions were statistically different (*p* < 0.05). Polarized iNOS^+^ macrophage cells with soluble DGEA are dramatically lower than iNOS^+^ macrophage cells without any presence of DGEA. Quantifying the graphs in Fig. 1(c) shows that iNOS^+^-activated macrophages are 0.5 ± 0.1 fraction of the total DAPI^+^ cells, whereas M1-activated macrophages in the presence of DGEA are 0.1 ± 0.1 fraction of the total DAPI^+^ cells. This corroborated our hypothesis that DGEA can act as an inhibitor in polarizing M1 macrophages.

Prior work within the field has also demonstrated that DGEA as well as other ECM-derived peptides can be used to manipulate cell function. The interaction between collagen and α2β1 integrin is considered vital for the osteoblastic differentiation of bone marrow cells. As shown by Mizuno et al., the addition of DGEA peptide to a culture of bone marrow cells encapsulated in type I collagen matrix gels, inhibited the expression of osteoblastic phenotype of bone marrow cells [13]. Previous work has also suggested DGEA to be a potential blocker for α2β1, particularly Luzak et al. demonstrated significant inhibition in platelet adhesion, as DGEA blocked surface receptors interacting with collagen [21]. Fishman et al. demonstrated DGEA to block adhesion of ovarian carcinoma cells to collagen [16], and Cha et al. [14] showed that the presence of DGEA affects macrophage response via α2β1 integrin binding. Thus, the reduction in iNOS^+^ cells upon culture with DGEA is within the context of established literature.

### Soluble delivery of DGEA in a 3D matrix of PEG hydrogels

**Figure 2:**
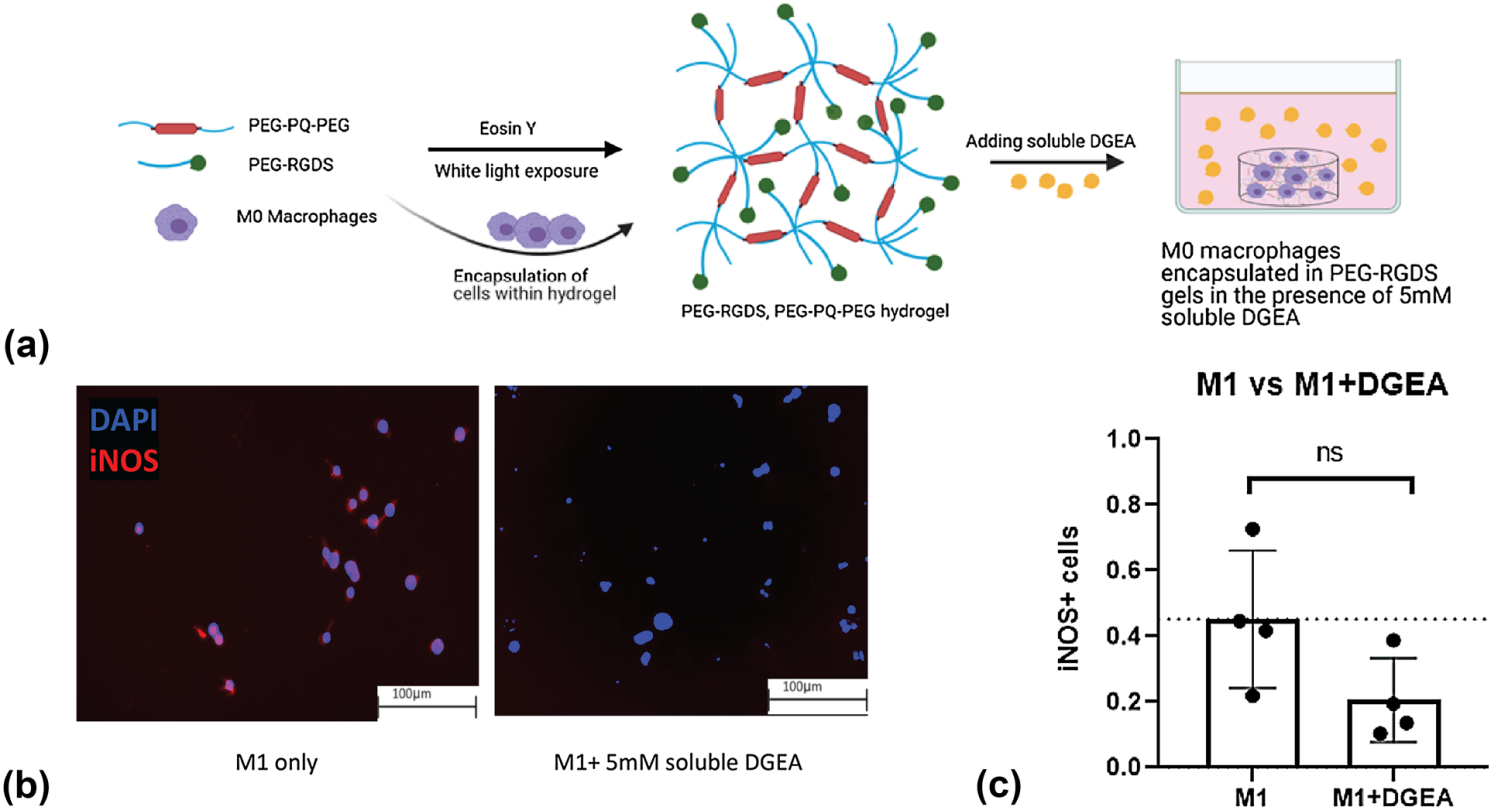
Soluble delivery of DGEA to cells encapsulated in 3D PEG hydrogels. (a) Soluble 5 mM DGEA peptide in control hydrogels. Raw 264.7 cells encapsulated within 5 μl droplet of control hydrogels. (b) iNOS and DAPI-stained images of M1 macrophages with and without the addition of 5 mM soluble DGEA. (c) iNOS^+^ cells were normalized to DAPI^+^ cells (*n* = 4). Data represent a student’s *t* test and significance is determined by *p* < 0.05.

Polyethylene glycol (PEG) is a synthetic polymer largely used as scaffolds in tissue engineering research due to its tunable nature [22]. In this work, we utilize acrylate-PEG-succinimidyl valerate (acryl-PEG-SVA). To immobilize the peptide, we react the peptide with the acryl-PEG-SVA. Via amine substitution, our product is acrylate-PEG-peptide. The acrylate group on the end of the acrylate-PEG-peptide chain allows for immobilization into the crosslinked hydrogel. While PEG is hydrophilic, these acrylate groups are hydrophobic in nature creating micelle-like centers in which free radicals rapidly propagate after initiation [23]. To further evaluate whether soluble delivery of DGEA had the same inhibitory effect on M1 macrophage polarization in a 3D microenvironment, we utilized an ECM-mimicking PEG-based hydrogel [24–26]. With PEG as its backbone, this hydrogel contained a cell-adhesive component RGDS (Arg-Gly-Asp-Ser) and an enzyme-cleavable component GGGPQGIWGQGK, abbreviated as PQ. This is referred to as the control hydrogel (Fig. 2(a)). While fibronectin derived RGDS provides sites for cell adhesion, PQ is a matrix metalloprotease (MMP-2/9)-sensitive peptide from the collagen chain that is cleaved in the presence of MMPs-2 and -9. This enzyme-specific cleavage allows for cell-mediated migration through the PEG-based hydrogel [27]. The incorporation of this enzyme-cleavable component mimics the natural ECM [28]. The PEG macromers, acrylate-PEG-RGDS, and acrylate-PEG-PQ-PEG-acrylate are mixed with cells, photoinitiator eosin Y, and *N*-Vinylpyrrolidone (NVP). Following white light exposure, free radicals are generated and propagate in the micelle-like acrylate centers, thus, allowing crosslinking and rapid polymerization of the hydrogel.

Raw 264.7 macrophages were encapsulated within the hydrogel. These quiescent M0 macrophages were allowed to equilibrate in the incubator for 24 h, after which M1 media was added to stimulate them towards the pro-inflammatory phenotype. At this time, we also dissolved 5 mM DGEA into the media and let the samples incubate for another 72 h. The samples were then fixed, stained, imaged, and analyzed. The immunofluorescent images represent DAPI-stained cells (in blue) and iNOS-stained cells (in red) (Fig. 2(b)). All 3D hydrogels were imaged using a Keyence BZ-X800 microscope, and all images represent 2D slices of an entire 3D Z-stack. The Student’s *t* test shown in Fig. 2(c) had similar trends when compared to Fig. 1(c); i.e., the number of iNOS^+^ cells in the presence of soluble DGEA was lower than the number of iNOS^+^ cells without any DGEA. Statistically, the data are non-significant (*p* > 0.05). This non-significance may be due to improper diffusion of the DGEA peptide into the hydrogel, thus, minimizing exposure of the encapsulated cells to DGEA treatment. However, observing similar trends in reduction of M1 macrophage suggested that DGEA can reduce M1 polarization via soluble delivery in a PEG hydrogel.

### Immobilizing DGEA in a PEG hydrogel and assessing conjugation

**Figure 3:**
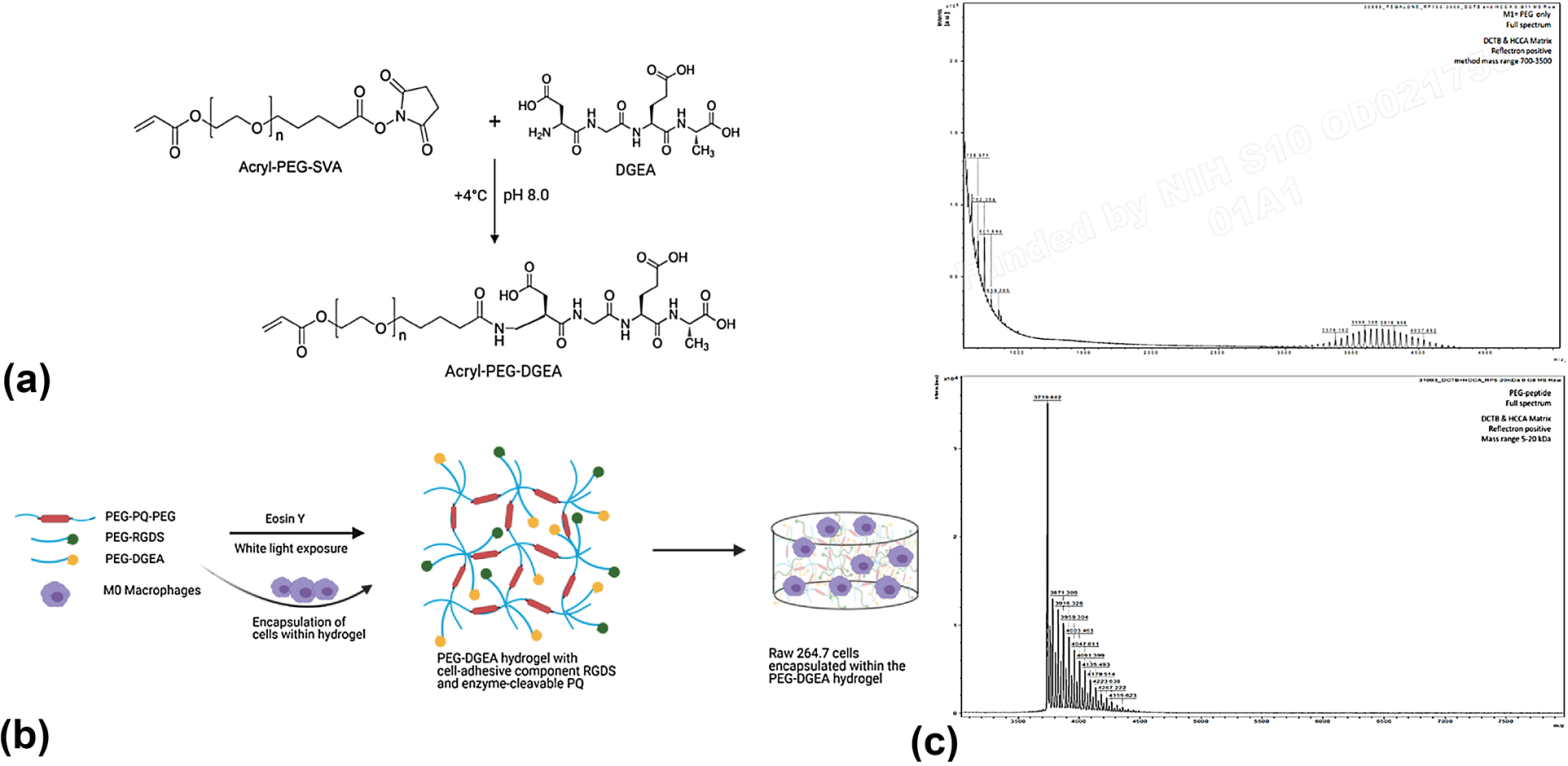
Immobilizing DGEA in PEG hydrogel and assessing conjugation efficiency. (a) Chemical structure of PEG-DGEA after crosslinking Acryl-PEG-SVA with DGEA. (b) A representation of the formation of experimental PEG-DGEA hydrogel with PEG-RGDS peptide and PEG-PQ-PEG. (c) MALDI-ToF analysis of PEG peptide only (top right) and PEG-DGEA (bottom right), to assess conjugation of PEG with peptide.

To design a biomaterial with ECM-mimicking characteristics, we immobilized DGEA in the hydrogel environment. The control hydrogel was an ECM-mimicking PEG hydrogel conjugated with RGDS, the cell-adhesive component, and PQ, the enzyme-cleavable component. Following previously described crosslinking chemistry, we designed a new biomaterial that incorporated DGEA in the control hydrogel, in the form of PEG-DGEA. This is a novel design as, to our knowledge, no previous studies have employed PEG-RGDS, PEG-PQ-PEG, and PEG-DGEA into a single hydrogel. The chemical formulations of the organic compounds are as depicted in Fig. 3(a). DGEA and other ECM-derived peptides such as laminin-derived IKVAV and YIGSR have been used covalently with PEG in the form of a biofunctionalized hydrogel for purposes such as encapsulating islets to promote cell viability [25] or investigating the effects of the peptides on valve interstitial cells [26].

MALDI-ToF was applied to monitor the conjugation of PEG with DGEA (Fig. 3(c)) [29, 30]. The *x*-axis represents the mass-to-charge ratio (m/z), while the y-axis represents intensity in fluorescence arbitrary units. The molecular weight (MW) of the PEG monomer (Acryl-PEG-SVA) is 3400 g/mol, (Fig. 3(c) top right) as is displayed by a dominant peak closer to the origin of *x*- and *y*-axes. The molecular weight of DGEA is 390.35 g/mol. The shift in peak from PEG to PEG-DGEA (Fig. 3(c) bottom right) is evident as a higher molecular weight of PEG-DGEA (MW = 3790.5 g/mol) weighs the curve pushing the peak further away from the origin of both axes. This suggests successful conjugation of PEG-DGEA. Cells were encapsulated in the PEG-DGEA hydrogel or control hydrogel as described in the following section.

### Immobilized DGEA in a PEG hydrogel platform has inhibitory effects on M1 macrophage polarization

**Figure 4:**
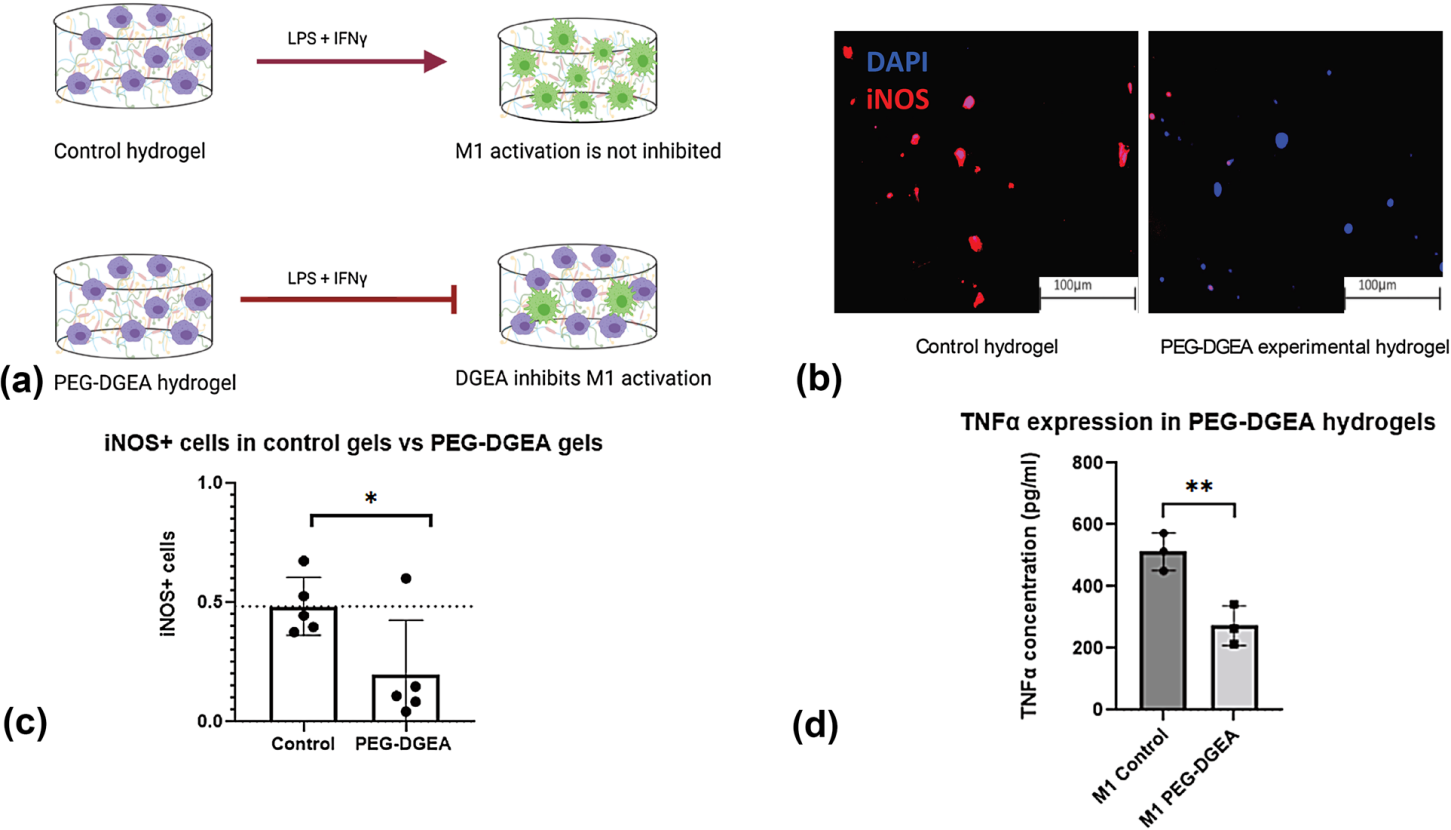
M0 macrophages were polarized to the M1 phenotype. Raw 264.7 cells were encapsulated in control and experimental hydrogels to test the behavior of DGEA on M1 macrophage polarization. (b) Immunofluorescent images of samples stained for DAPI and iNOS. M1 macrophages encapsulated in (left) control hydrogels (3.5 mM RGDS, 5% PQ), and (right) experimental hydrogels (5 mM DGEA, 3.5 mM RGDS, 5% PQ). (c) Student’s *t* test was utilized to assess the number of iNOS^+^ cells/DAPI^+^ cells in both conditions (*n* = 5). Significance is depicted by (*p < 0.05). (d) ELISA analysis of TNFα expression in the conditioned media of M1 macrophages encapsulated in PEG-DGEA hydrogels (**p < 0.01).

Next, we assessed M1 response in a 3D environment with immobilized DGEA. A study by Mehta et al. highlighted the role of DGEA immobilized in alginate hydrogels to induce an osteogenic phenotype in mesenchymal stem cells [31]. Employing the PEG-DGEA hydrogel, as described in the previous section, and control hydrogel as the experimental and control groups, respectively, M0 macrophages were encapsulated in each gel (Fig. 4(a)). M1 media was added 24 h post-encapsulation to stimulate M0 macrophages towards the M1 phenotype. 72 h following the addition of M1 media, cells were fixed, stained, and analyzed. The immunostaining of cells using iNOS and DAPI is represented in Fig. 4(b). M1 macrophages encapsulated in the control hydrogels had 0.5 ± 0.1 iNOS^+^ cells per total DAPI^+^ cells. On the other hand, M1 macrophages in PEG-DGEA hydrogels had a ratio of less than 0.2 ± 0.1 of iNOS^+^ cells/DAPI^+^ cells. Student’s *t* tests were performed to evaluate if the data were statistically significant (Fig. 4(c)). The results of the statistical analysis mirrored the visual observations from images of stained samples. The data are statistically different for both conditions. Further, conditioned media from the encapsulated M1 macrophages was collected for ELISA analysis of soluble cytokine secretion. TNFα is a pro-inflammatory cytokine robustly secreted by M1 macrophages [32]. Figure 4(d) demonstrates a significant reduction in the expression of TNFα by M1 macrophages encapsulated in PEG-DGEA hydrogels for 72 h. This reduction contributes to the claim that in the 3D in vitro environment of PEG-DGEA hydrogels, M1 macrophages can be less inflammatory. Our studies represent that the novel PEG-DGEA hydrogel has inhibitory effects on M1 macrophage polarization.

### PEG-DGEA has inhibitory effects on polarization of human-derived M1 macrophages

**Figure 5:**
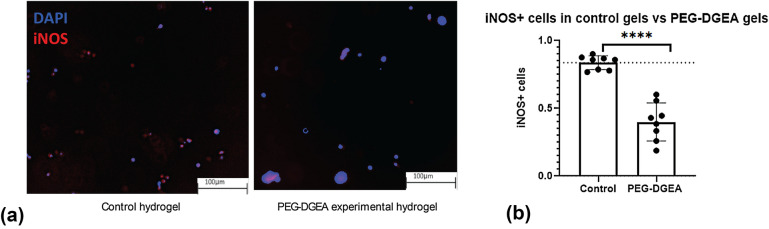
Macrophages derived from a healthy donor were encapsulated in control and experimental hydrogels. (a) Immunofluorescent images of samples stained for DAPI (blue) and iNOS (red). M1 macrophages encapsulated in (left) control hydrogels, and (right) PEG-DGEA experimental hydrogels. (b) Student’s *t* test was utilized to assess number of iNOS^+^ cells/DAPI^+^ cells in both conditions (*n* = 8). Significance is depicted by (*p* < 0.05).

To assess if PEG-DGEA’s inhibitory effects on iNOS expression in murine macrophages could be translated to human macrophages, we isolated monocytes from a healthy human blood sample. The monocytes were converted to macrophages on addition of RPMI-1640 supplementary media. These macrophages were encapsulated in control and PEG-DGEA hydrogels as previously described. Following encapsulation, human macrophages were stimulated from M0 to the M1 phenotype by adding LPS and IFNγ in the RPMI medium. Samples were stained for iNOS and DAPI for all conditions (Fig. 5(a)). A Student’s *t* test revealed a significant difference between iNOS^+^ cells for control hydrogels versus iNOS^+^ cells for experimental hydrogels. We observe 0.85 ± 0.1 iNOS^+^cells per total DAPI^+^cells in the control conditions, and a 0.4 ± 0.1 iNOS^+^cells per total DAPI^+^cells in the PEG-DGEA hydrogels. These results suggest effective human translation of the PEG-DGEA hydrogel to control inflammatory conditions by manipulating the M1 macrophage phenotype.

### Comparison between models

To interrogate the hypothesis of DGEA influencing macrophage polarization, we first delivered soluble DGEA in a 2D culture of Raw 264.7 macrophages. Analyzing this study demonstrated that DGEA inhibits iNOS expression in M1 macrophages, thus, reducing M1 polarization. Reduction of iNOS following 2D soluble delivery prompted assessment of soluble effects of DGEA in 3D. Thus, we conducted a soluble delivery study with a 3D control PEG hydrogel. We dissolved 5 mM DGEA peptide in the media to assess macrophage response via iNOS expression. As previously explained, the control hydrogel contained RGDS and PQ. The soluble delivery of DGEA in 3D did not statistically inhibit iNOS expression in M1 macrophages. However, the trend displays that soluble delivery of DGEA in 3D did reduce iNOS expression (Fig. 2(c)). We hypothesize that diffusion of the soluble-delivered DGEA peptide through the crosslinked hydrogel was insufficient to significantly alter function of encapsulated macrophages. Furthermore, even though PEG hydrogels are known to allow for diffusion of nutrients, the literature suggests that the inert state of PEG hydrogels hinders therapeutic efficacy of locally delivered soluble factors targeted to the encapsulated cells [33]. Previous studies have highlighted the importance of immobilizing peptides or therapeutics in PEG hydrogels for optimum drug availability and enhanced interactions with the encapsulated cells. To design a hydrogel in which DGEA is immobilized and covalently crosslinked, we conjugated PEG to DGEA for grafting into the PEG hydrogel. This new hydrogel constituted of PEG-DGEA, PEG-RGDS, and PEG-PQ-PEG. This novel hydrogel exposed M1 macrophages to the immobilized DGEA peptide and reduced iNOS expression.

## Conclusions and future work

Utilizing ECM-derived peptides to design immune-informed biomaterials can inform clinical translation for therapeutics in regenerative medicine. The tunable properties of such biomaterials allow researchers to manipulate cell functions such as preventing inflammation, controlling fibrosis, and promoting tissue healing. This work highlights the development of a novel biomaterial to inhibit pro-inflammatory macrophage polarization. Future studies will focus on the pathways affected by the DGEA peptide in relation to inflammation by gene expression analysis and soluble protein secretion. There is also scope to explore integrin-mediated interactions in cell binding and migration in human cells with consideration of age and sex. Our work is a proof of concept that DGEA can indeed play a role in inhibiting M1 macrophage polarization.

## Methods

### Cell culture and maintenance

**Figure 6:**
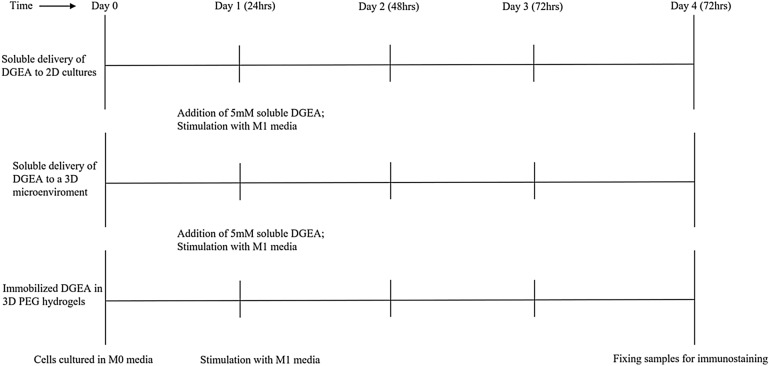
Timeline of experimental design for all modes of DGEA assessment on M1 macrophage phenotype.

The cell line Raw 264.7, derived from BALB/c mice, was obtained from ATCC. The cells were cultured in Dulbecco's Modified Eagle's Medium (DMEM) (Corning, Corning, NY) supplemented with 10% fetal bovine serum (FBS) (Atlanta Biologicals, Lawrenceville, GA), 100 IU penicillin, and 100 μg/ml streptomycin (Corning). For the purposes of this paper, we label this M0 media. To stimulate the cells towards the M1 phenotype, 10 ng/ml of IFNγ (Prospec, East Brunswick, NJ) along with 100 ng/ml of LPS (Santa Cruz Biotechnology, Dallas, TX) was added to M0 media. This is referred to as M1 media. Cells were stimulated to the M1 phenotype 24 h post-seeding on a 24-well tissue culture polystyrene (TCP) plate. M0 macrophages were also cultured over the same time periods, resulting in two groups through 72 h (M0 and M1) (refer to Fig. 6 for experimental design). All cells were maintained at 37 °C in 5% CO_2_.

Peripheral blood was obtained in ethylenediaminetetraacetic (EDTA) vacutainer collection tubes from a healthy Caucasian female donor (#IRB202001085), and peripheral blood mononuclear cells (PBMCs) were isolated from whole blood by Ficoll gradient centrifugation. 35 ml blood was diluted 1:1 in calcium/magnesium-free phosphate-buffered saline (PBS), slowly layered over 15 ml Ficoll-Paque (GE Healthcare, Piscataway, NJ), and then centrifuged at 400 g for 30 min at room temperature with brakes off. PBMCs were collected and washed twice in PBS by centrifuging at 300 g for 10 min. PMBCs were then resuspended in a cell suspension buffer consisting of PBS pH 7.2, 0.5% bovine serum albumin (Fisher Scientific), and 2 mM EDTA calcium disodium salt hydrate (TCI America). Monocytes were isolated from PBMCs via magnetic activated cell sorting using a Pan-Monocyte Isolation kit (Milentyi Biotec). Isolated monocytes were collected and washed in 1 ml RPMI-1640 (Gibco, Grand Island, NY). Monocytes were plated on a 6-well tissue culture dish at a density of 5.88 × 10^6^ cells/ml. To differentiate macrophages from monocytes, RPMI-1640 was supplemented with 2 mM l-glutamine (Gibco), 100 U/ml penicillin (Corning), 100 μg/ml streptomycin (Corning), 0.1 mM sodium pyruvate (Gibco), 1% non-essential amino acids (Gibco), 50 μM 2-mercaptoethanol (Gibco), and 10% fetal bovine serum (Atlanta Biologicals) and 20 ng/ml macrophage-colony stimulating factor (M-CSF) (Life Technologies, Carlsbad, CA). Media with M-CSF was changed every 48 h post-seeding. All cells were maintained at 37 °C in 5% CO_2_. After 5 days of incubation, cells in the M0 state were encapsulated in control and experimental hydrogels, as described in the subsequent sections. M1 media was added to the well plates to stimulate macrophages towards the M1 phenotype. This M1 media contained RPMI-1640 with all supplements previously listed, except M-CSF. Instead, 10 ng/ml IFNγ was added along with 100 ng/ml of LPS. Cells were allowed to incubate for 72 h for subsequent experiments.

### PEG hydrogel fabrication

DGEA (Asp-Gly-Glu-Ala) peptide (obtained from Genscript) was conjugated to PEG by amine substitution reaction of the ECM-derived peptide with acrylate-(poly (ethylene glycol) (PEG)-succinimidyl valerate (SVA) (acrylate-PEG-SVA; Laysan Bio Inc., Arab, AL). A 1.2:1 molar ratio of DGEA peptide to acrylate-PEG-SVA was mixed in 20 mM (*N*‐(2‐hydroxyethyl)piperazine‐*N*′‐(4‐butanesulfonic acid)) (HEPBS) buffer with 100 mM NaCl, 2 mM CaCl2, and 2 mM MgCl_2_ at pH 8.5 (referred to as protein conjugation buffer) [34]. The pH of this mixture was then titrated to 8.0 and reacted overnight (16 h) at 4 ℃ under constant agitation. The final product (acrylate‐PEG‐DGEA) was then dialyzed (3.5 kDa molecular weight cut-off MWCO regenerated cellulose; Spectrum Laboratories), lyophilized, and stored at − 80 °C (Labconco, Kansas City, MO) until use. The same steps were repeated for crosslinking the cell-adhesive component RGDS (Arg-Gly-Asp-Ser), with PEG to form PEG-RGDS with the molar ratio 1.2:1. A 1:2 molar ratio of PQ (GGGPQGIWGQGK) peptide to acrylate‐PEG‐SVA was used for the synthesis of the diacrylate polymer PEG-PQ-PEG. The only difference in the PEG-PQ-PEG conjugation is the molar ratio as well as the dialysis cut-off, and the final product was dialyzed at 6–8k MWCO.

The molecular weights of each peptide used are displayed in Table 1.

**TABLE 1:** Summarized molecular weights and molar ratios of each peptide used in this work before and after conjugation with PEG.

Polymer modification with peptides	Molecular weight (g/mol)	Molar ratio (PEG peptide)	Conjugated product
Acryl-PEG-SVA	3400	–	–
RGDS	433.42	1:1.2	PEG-RGDS
PQ	1141	2:1	PEG-PQ-PEG
DGEA	390.35	1:1.2	PEG-DGEA

Successful conjugation of the DGEA peptide with PEG was confirmed via matrix-assisted laser desorption/ionization-time-of-flight mass spectrometry (MALDI-ToF) (funded by NIH S10 OD021758-01A1). MALDI was performed on Acryl-PEG-SVA, PEG-RGDS, PEG-PQ-PEG, and PEG-DGEA. MALDI matrix DCTB only, HCCA only, and HCCA & DCTB were prepared in a 1:1 ratio of acetonitrile:water with 0.1% Trifluoroacetic acid (TFA). Sample solution was mixed with 3 different MALDI matrices in 1:1 (v/v) ratio. The sample-matrix mixture was spotted on the MALDI plate (1 µl) for analysis.

### Encapsulation of cells within PEG hydrogels

After conjugation, the hydrogels were split into control groups and experimental groups wherein the control group was a PEG-RGDS and PEG-PQ-PEG hydrogel. The experimental condition was a hydrogel construct of PEG-RGDS, PEG-PQ-PEG, as well as PEG-DGEA. To form hydrogels, the polymers (2.5% PEG‐PQ‐PEG and 3.5 mM PEG‐RGDS) were dissolved in a HEPES‐buffered saline (HBS; 10 mM HEPES; and 100 mM NaCl at pH 7.4) with 1.5% triethanolamine (TEOA; Sigma), 10 μM eosin Y and 0.35% (v/v) *N*-vinyl-pyrrolidone (NVP; Sigma) at pH 8.3. Raw 264.7 macrophages were encapsulated in the hydrogels at 50,000 cells per gel. A 5 μl droplet of the cell‐polymer suspension was placed on top of a 385um PDMS slab, with two PDMS spacers to allow formation of a spheroid 3D gel [34]. A methacrylate‐modified glass coverslip, bearing groups that permit covalent bonding with the hydrogel, was placed on top of the 5 μl droplet. This was done for ease of handling of the delicate hydrogel and ease of culturing in 24-well plates. The cell-polymer suspension in between the PDMS spacers and the coverslip was exposed to UV light for 60 s to allow the droplet to solidify into a hydrogel construct. The coverslip, with the hydrogel facing up, was planted in each well to which media was added in order to supply nutrients to the cells encapsulated within.

### Soluble delivery of DGEA to encapsulated cells in PEG hydrogels

For soluble delivery of the DGEA peptide in a 3D matrix, 3.5 mM PEG-RGDS and 5% PEG-PQ-PEG hydrogels were made as the control hydrogel. 1 ml M0 media was added to each well of the 24-well plate containing the hydrogels and incubated at 37 °C in 5% CO_2_. 24 h post-encapsulation, M0 media was aspirated and the cells were rinsed with PBS. Wells were split into control and experimental groups. M0 media was added to the control wells, and M1-stimulating media was added to the experimental wells (refer to Fig. 6). Media changes occurred at 24 h post‐encapsulation and subsequently every 48 h afterwards. 5 mM DGEA was dissolved into both M0 and M1 media at 0 mM and 5 mM to assess impact of soluble DGEA on cells in a 3D matrix.

### Encapsulating cells in PEG hydrogels with immobilized DGEA

Similarly, for assessing M1 macrophage response to immobilized DGEA, Raw 264.7 cells were encapsulated in a 3D matrix of 5 mM PEG-DGEA, 3.5 mM PEG-RGDS, and 5% PEG-PQ-PEG gels. This has been defined as the experimental hydrogel. 1 ml M0 media was added to each well of the 24-well plate containing the hydrogels. All gels were cultured at 37 °C in 5% CO_2_. 24 h post-encapsulation, M0 media was aspirated and the cells were rinsed with PBS. M1 media was added to all experimental conditions, and control groups were continually cultured in M0 media. Media changes occurred at 24 h post‐encapsulation and subsequently every 48 h afterwards (refer to Fig. 6). The same steps were followed to encapsulate human macrophages in control and experimental hydrogels at a density of 6 million cells/ml (30,000 cells per gel).

### Immunostaining

Immunostaining assays were carried out to analyze the expression levels of iNOS (M1 surrogate marker) and DAPI (nuclei marker) on Raw 264.7 macrophages and human macrophages. Raw 264.7 cells were seeded at 10 million cells/ml onto 24-well plates (50,000 cells per gel). Human macrophages were seeded within the control and experimental hydrogels at 6 million cells/ml (30,000 cells per gel). After being cultured for 72 h post-addition of M1 media, the cells were fixed with 4% paraformaldehyde for 45 min at room temperature and then washed 3 times with tris-buffered saline (TBS). Gels were then permeabilized in 0.25% Triton-X for 45 min, rinsed with TBS 4 times, followed by blocking overnight in 5% donkey serum (DS) at 4 ℃. Rinses after blocking took place 3 times in TBS for 5 min each.

Following blocking, gels were incubated in the primary antibody iNOS (M1 marker) (Rabbit Anti-Mouse Polyclonal Antibody, Invitrogen) at a dilution of 1:200 in 0.5% DS at 4℃ overnight. Following primary incubation, gels were rinsed 5 times with TBS + 0.01% Tween for 90–120 min. The 5th rinse was left overnight at 4 ℃. On the consecutive morning, the 6th and final rinse was in TBS alone without the presence of Tween. Gels were then incubated overnight at 4 ℃ with secondary antibody AlexaFluor 555 (Donkey Anti-Rabbit, Life Technologies) at 1:100, following an hour-long rinse with TBS the next day. Cell nuclei were stained with 2 μM 4′,6-diamidino-2-phenylindole (DAPI; nuclear marker). Samples were rinsed twice with TBS for 5 min each before imaging.

### Imaging and image analysis

Cells were stimulated with M1 media 24 h post-seeding and post-encapsulation. In all studies, the effects of DGEA on the cells were assessed 72 h post-stimulation with M1 media by immunocytochemistry (ICC). Cells in the soluble studies and encapsulated in gels were imaged using the Keyence BZ-X800 microscope, 72 h post-stimulation to the M1 phenotype.

Images were quantified based on the number of iNOS^+^ cells, normalized to DAPI^+^ cells for each condition. Images were analyzed using the ‘automated cell counting of single color image’ feature on ImageJ (NIH) software after a randomized, unbiased selection of images. All images were turned to 8-bit grayscale and thresholded to highlight all the cells to be counted. Total cell count for each image, recorded as iNOS^+^ and DAPI^+^ cells, were saved in Microsoft Excel and then exported to GraphPad Prism for further statistical analysis.

### Enzyme linked immunosorbent assay (ELISA) for TNFα expression

Raw 264.7 cells were encapsulated in control and PEG-DGEA hydrogels to assess differences in TNFα expression due to immobilized DGEA. Post-treatment with M1 media, conditioned media, or cell supernatant was collected. The concentrations of TNFα were measured by utilizing a mouse ELISA kit (RayBiotech) and following manufacturer’s instructions. Student's *t* test was used to determine differences in cytokine expression between the treatment groups.

### Statistical analyses

Throughout the experiments, i.e., soluble DGEA delivery in a 2D environment on TCP, soluble DGEA delivery in a 3D matrix, as well as immobilized DGEA in a 3D matrix, the total cell count of iNOS^+^ and DAPI^+^ cells was exported into GraphPad Prism 8.4.1 (La Jolla, CA). iNOS^+^ cells were normalized to DAPI^+^ cells. M1 macrophages across all experiments, with and without the presence of DGEA, were paired and an independent Student’s *t* test was employed to investigate the difference between means. The number of samples ranged from 4 to 8 per condition in all of the analyses. Statistical significance is reported as *p* < 0.05. The confidence interval is reported as 95%. Results are presented as the mean with ± standard deviation.

## Data Availability

The datasets generated during and/or analyzed during the current study are available from the corresponding author on reasonable request (E.M).

## References

[1] Chen L (2018). Inflammatory responses and inflammation-associated diseases in organs. Oncotarget.

[2] R. Pahwa and I. Jialal, Chronic inflammation - StatPearls - NCBI Bookshelf. *Stat**Pearls*. (2019). 29630225

[3] Ferrante CJ, Leibovich SJ (2012). Regulation of macrophage polarization and wound healing. Adv. Wound Care.

[4] Martinez FO, Gordon S (2014). The M1 and M2 paradigm of macrophage activation: Time for reassessment. F1000Prime Rep..

[5] Atri C, Guerfali FZ, Laouini D (2018). Role of human macrophage polarization in inflammation during infectious diseases. Int. J. Mol. Sci..

[6] Duque GA, Descoteaux A (2014). Macrophage cytokines: Involvement in immunity and infectious diseases. Front. Immunol..

[7] Rath M, Müller I, Kropf P, Closs EI, Munder M (2014). Metabolism via arginase or nitric oxide synthase: Two competing arginine pathways in macrophages. Front. Immunol..

[8] Parisi L (2018). Macrophage polarization in chronic inflammatory diseases: Killers or builders?. J. Immunol. Res..

[9] Krzyszczyk P, Schloss R, Palmer A, Berthiaume F (2018). The role of macrophages in acute and chronic wound healing and interventions to promote pro-wound healing phenotypes. Front. Physiol..

[10] Vigier S, Fülöp T (2016). Exploring the Extracellular Matrix to Create Biomaterials. Composition and Function of the Extracellular Matrix in the Human Body.

[11] Staatz WD, Fok KF, Zutter MM, Adams SP, Rodriguez BA, Santoro SA (1991). Identification of a tetrapeptide recognition sequence for the α2β1 integrin in collagen. J. Biol. Chem..

[12] Popov C (2011). Integrins α2β1 and α11β1 regulate the survival of mesenchymal stem cells on collagen I. Cell Death Dis..

[13] Mizuno M, Fujisawa R, Kuboki Y (2000). Type I collagen-induced osteoblastic differentiation of bone-marrow cells mediated by collagen-α2β1 integrin interaction. J. Cell. Physiol..

[14] Cha BH (2017). Integrin-mediated interactions control macrophage polarization in 3D hydrogels. Adv. Healthc. Mater..

[15] Liu Y, Segura T (2020). Biomaterials-mediated regulation of macrophage cell fate. Front. Bioeng. Biotechnol..

[16] Fishman DA (1998). Metastatic dissemination of human ovarian epithelial carcinoma is promoted by α2β1-integrin-mediated interaction with type I collagen. Invasion Metastasis.

[17] Wu Y, Grande-Allen KJ, West JL (2016). Adhesive peptide sequences regulate valve interstitial cell adhesion, phenotype and extracellular matrix deposition. Cell. Mol. Bioeng..

[18] Patel D, Sharma S, Bryant SJ (2018). Tenocyte attachment to collagen mimetic peptides increase mechano-sensitivity to shear and tension. Orthop. Proc..

[19] Reddy J, Gerasimov M, Griffith M (2019). Functional modification of collagen like peptides for cellular specificity and function. Investig. Ophthalmol. Vis. Sci..

[20] Yoo SY, Kobayashi M, Lee PP, Lee SW (2011). Early osteogenic differentiation of mouse preosteoblasts induced by collagen-derived DGEA-peptide on nanofibrous phage tissue matrices. Biomacromol.

[21] Luzak B, Golanski J, Rozalski M, Boncler MA, Watala C (2003). Inhibition of collagen-induced platelet reactivity by DGEA peptide Circled white star. Acta Biochim. Pol..

[22] Peters EB, Christoforou N, Leong KW, Truskey GA, West JL (2016). Poly(ethylene glycol) hydrogel scaffolds containing cell-adhesive and protease-sensitive peptides support microvessel formation by endothelial progenitor cells. Cell. Mol. Bioeng..

[23] Hern DL, Hubbell JA (1998). Incorporation of adhesion peptides into nonadhesive hydrogels useful for tissue resurfacing. J. Biomed. Mater. Res..

[24] E. M. Moore, Investigating the roles of macrophages in vessel development utilizing poly(ethylene glycol) hydrogels. (2018).

[25] Weber LM, Hayda KN, Haskins K, Anseth KS (2007). The effects of cell–matrix interactions on encapsulated β-cell function within hydrogels functionalized with matrix-derived adhesive peptides. Biomaterials.

[26] Wu Y, Jane Grande-Allen K, West JL (2016). Adhesive peptide sequences regulate valve interstitial cell adhesion, phenotype and extracellular matrix deposition. Cell. Mol. Bioeng..

[27] Moon JJ (2010). Biomimetic hydrogels with pro-angiogenic properties. Biomaterials.

[28] West JL, Hubbell JA (1999). Polymeric biomaterials with degradation sites for proteases involved in cell migration. Macromolecules.

[29] Zhou G, Khan F, Dai Q, Sylvester JE, Kron SJ (2012). Photocleavable peptide-oligonucleotide conjugates for protein kinase assays by MALDI-TOF MS. Mol. Biosyst..

[30] Kemptner J, Marchetti Deschmann M, Siekmann J, Turecek PL, Schwarz HP, Allmaier G (2010). GEMMA and MALDI-TOF MS of reactive PEGs for pharmaceutical applications. J. Pharm. Biomed. Anal..

[31] Mehta M, Madl CM, Lee S, Duda GN, Mooney DJ (2015). The collagen I mimetic peptide DGEA enhances an osteogenic phenotype in mesenchymal stem cells when presented from cell-encapsulating hydrogels. J. Biomed. Mater. Res. A.

[32] Lv R, Bao Q, Li Y (2017). Regulation of M1-type and M2-type macrophage polarization in RAW264.7 cells by galectin-9. Mol. Med. Rep..

[33] Lin C-C, Anseth KS (2008). PEG hydrogels for the controlled release of biomolecules in regenerative medicine. Pharm. Res..

[34] Moore EM, Ying G, West JL (2017). Macrophages Influence Vessel Formation in 3D Bioactive Hydrogels. Adv. Biosyst..

